# Formulation of Bio-Based Washing Agent and Its Application for Removal of Petroleum Hydrocarbons From Drill Cuttings Before Bioremediation

**DOI:** 10.3389/fbioe.2020.00961

**Published:** 2020-08-11

**Authors:** Noulkamol Arpornpong, Rattiya Padungpol, Nichakorn Khondee, Chantra Tongcumpou, Suwat Soonglerdsongpha, Komkrit Suttiponparnit, Ekawan Luepromchai

**Affiliations:** ^1^Department of Natural Resources and Environment, Faculty of Agriculture, Natural Resources and Environment, Naresuan University, Phitsanulok, Thailand; ^2^Microbial Technology for Marine Pollution Treatment Research Unit, Department of Microbiology, Faculty of Science, Chulalongkorn University, Bangkok, Thailand; ^3^Environmental Research Institute, Chulalongkorn University, Bangkok, Thailand; ^4^Research Program on Remediation Technologies for Petroleum Contamination, Center of Excellence on Hazardous Substance Management, Chulalongkorn University, Bangkok, Thailand; ^5^Environmental Technology Research Department, Innovation Institute, PTT Public Company Limited, Bangkok, Thailand

**Keywords:** drilling waste, soil washing, biosurfactants, polyolefin biodegradation, sequential treatment

## Abstract

Drill cuttings from petroleum exploration and production sites can cause diverse environmental problems. Total petroleum hydrocarbons (TPHs) are a major pollutant from the use of polyolefin-based mud. As an alternative to incineration, this study investigated the application of surfactant-enhanced washing technology prior to bioremediation. The washing step was necessary because the initial TPH concentrations were quite high at approximately 15% (w/w). Washing agents were formulated by varying the concentration of lipopeptide biosurfactant (in foamate or cell-free broth), Dehydol LS7TH (fatty alcohol ethoxylate 7EO, oleochemical surfactant) and butanol (as a lipophilic linker) at different salinities. The most efficient formula produced a Winsor Type I microemulsion (oil-in-water microemulsion) with polyolefin and contained only 20% (v/v) foamate and 2% (v/v) Dehydol LS7TH in water. Due to the synergistic behavior between the anionic lipopeptides and non-ionic Dehydol LS7TH, the formula efficiently removed 92% of the TPHs from the drill cuttings when applied in a jar test. To reduce the cost, the concentrations of each surfactant should be reduced; thus, the formula was optimized by the simplex lattice mixture design. In addition, cell-free broth, at a pH of 10, containing 3.0 g/L lipopeptides was applied instead of foamate because it was easy to prepare. The optimized formula removed 81.2% of the TPHs and contained 72.0% cell-free broth and 1.4% Dehydol LS7TH in water. A 20-kg soil washing system was later tested where the petroleum removal efficiency decreased to 70.7% due to polyolefin redeposition during separation of the washing solution. The remaining TPHs (4.5%) in the washed drilled cuttings were further degraded by a mixture of *Marinobacter salsuginis* RK5, *Microbacterium saccharophilum* RK15 and *Gordonia amicalis* JC11. To promote TPH biodegradation, biochar and fertilizer were applied along with bacterial consortia in a microcosm experiment. After 49-day incubation, the TPHs were reduced to 0.9% by both physical and biological mechanisms, while the TPHs in the unamended samples remained unaffected. With the use of the formulated bio-based washing agent and bioremediation approach, the on-site treatment of drill cuttings could be conducted with an acceptable cost and low environmental impacts.

## Introduction

Drilling waste from petroleum exploration and production sites can cause environmental problems due to the presence of petroleum hydrocarbons, heavy metals and inorganic salts in the drill cuttings (Sharif et al., 2017). The management options for drilling waste include incineration, thermal desorption, disposal in landfills, reuse in construction without prior treatment, stabilization/solidification, surfactant-enhanced washing, bioremediation and phytoremediation (Leonard and Stegemann, 2010; Ball et al., 2011; Fan et al., 2014; Kogbara et al., 2017; Liu et al., 2019). In Thailand, onshore petroleum production sites usually adopt synthetic based mud during operation and later transport the drill cuttings to waste management companies for incineration. The disadvantages of the current treatment options are their high cost, high energy use, high time consumption and notable environmental unsustainability (Leonard and Stegemann, 2010). Consequently, this study aimed to develop an alternative on-site treatment by formulating a bio-based washing agent and then applying it to reduce the concentration of petroleum hydrocarbons in drilling waste prior to bioremediation.

Petroleum hydrocarbons are the major contaminants in drilling waste with concentrations ranging from 1.5–15% (w/w). For example, 15,300 and 17,125 mg/kg of TPHs were present in drilling wastes from an active drilling operation in Sichuan, China and at a disposal facility in the Niger Delta region, Nigeria, respectively (Fan et al., 2014; Kogbara et al., 2017). Leonard and Stegemann (2010) reported a total petroleum hydrocarbon (TPH) concentration of 66,700 mg/kg in an unidentified onshore drilling operation. In addition, Fernandez et al. (2008) investigated a drilling waste sample that had been stored for 20–30 years in open cesspits in Tabasco, Mexico and measured a high TPH concentration of 135,400 mg/kg. This information suggests that the indigenous microorganisms in drill cuttings have a low TPH-degrading activity and that natural attenuation is not an appropriate option for drilling waste treatment. Bioremediation of oil-based cuttings, such as by composting, biopiling, slurry bioreactors and phytoremediation, has been performed as a cost-effective treatment; however, TPH biodegradation occurs slowly and may require up to 12 months for treatment of waste with high initial petroleum concentrations (Alavi et al., 2014; Fan et al., 2014; Chaîneau et al., 1995).

Surfactant-enhanced washing technology using either synthetic surfactants or biosurfactants has been considered an effective and rapid technique for the removal of petroleum hydrocarbons from soils and contaminated sites (Trellu et al., 2016; Befkadu and Chen, 2018). A single type of surfactant is usually applied as the washing agent; for example, Fernandez et al. (2008) found that 4% SDS (an anionic surfactant) and 1% ENP (a non-ionic surfactant) could remove 55.7% and 52.2%, respectively of the TPHs (at 13.5%) from drill cuttings stored for 20 to 30 years. Yan et al. (2011) used 0.03% rhamnolipid to remove 83% of 8.5% (w/w) petroleum in washed drill cuttings from the Liaohe oilfield in China. Recently, mixtures of anionic and non-ionic surfactants have been applied as their synergistic behavior can potentially increase pollutant washing efficiency. Shi et al. (2015) reported that a mixture of Triton X-100 and SDS increased the solubilization of polycyclic aromatic hydrocarbons (PAHs) and effectively removed PAHs from highly contaminated soil collected from an abandoned coke oven plant in China.

Another potential washing agent is a surfactant-based microemulsion, which can be formulated by mixing different surfactants in water or saline water and is based on the experimental phase behavior of a surfactant-oil-water system (Winsor Type region). The removal of petroleum usually occurs via two mechanisms: solubilization via a Winsor Type I microemulsion and mobilization via a Winsor Type III microemulsion (Javanbakht and Goual, 2016). Microemulsion-based washing agents can be achieved with a low surfactant amount; thus, the application of microemulsions could reduce the potential risk of new contaminants being released into the environment and ensure the economic practicality of the washing process (Arpornpong et al., 2018). In addition, the enhancement of petroleum solubility by microemulsions is linearly proportional to the concentration of microemulsion, while conventional surfactants promote solubilization of petroleum only in the vicinity of its critical micelle concentration (CMC) (Lau et al., 2014).

In this study, bio-based washing agents were formulated by investigating the microemulsion phase behavior of polyolefin, the major component in synthetic-based mud, and solutions of mixed bio-based surfactants at different salinities. Lipopeptides from *Bacillus subtilis* GY19 and Dehydol LS7TH, a fatty alcohol ethoxylate oleochemical surfactant, were selected because they are bio-based surfactants and have been applied to solubilize hydrophobic compounds such as crude petroleum and vegetable oil (Rongsayamanont et al., 2017; Arpornpong et al., 2018). The addition of butanol as a lipophilic linker was also investigated. Nguyen and Sabatini (2009) reported that increasing the surfactant/linker concentration in biosurfactant-based microemulsions enhances the lipophilicity of surfactant aggregates and leads to a lower salinity requirement in the microemulsion system. The TPH removal efficiency of bio-based washing agents was determined with drill cuttings in jar tests as well as a scale-up washing system. The washed drilled cuttings were further remediated by adding a mixture of *Marinobacter salsuginis* RK5, *Microbacterium saccharophilum* RK15, and *Gordonia amicalis* JC11, which are locally isolated petroleum-degrading bacteria. Compared to a single bioremediation or washing treatment, the two-stage remedial system can reduce treatment time and increase treatment efficiency (Yan et al., 2011). To promote TPH biodegradation, biochar and fertilizer were applied along with mixed bacteria to the washed drilled cuttings. Biochar can improve soil fertility and hydraulic properties as well as enhance contaminant immobilization and transformation (Lim et al., 2016; Zhu et al., 2017), while fertilizer can enhance the degradation potential of bacterial populations (Tyagi et al., 2011). With the use of the newly formulated bio-based washing agent and bioremediation approach, the on-site treatment of drill cuttings can become feasible.

## Materials and Methods

### Drill Cuttings, Surfactants, Bacteria and Chemicals

The surfactants studied in this work are classified into two main groups: biosurfactants and synthetic surfactants. Table 1 lists the properties of all the surfactants used in this study. The lipopeptide biosurfactant (anionic) was produced by chitosan-immobilized *Bacillus* sp. GY19 using waste glycerol and palm oil as substrates according to Khondee et al. (2015). The production medium was passed through a stainless steel screener (<0.5 mm), and bacterial cells were removed from the culture medium by centrifugation at 8,000 rpm for 20 min. Cell-free broth was sterilized by autoclaving at 121°C for 15 min. Lipopeptides as foamate were separated and concentrated following the foam fractionation method of Khondee et al. (2015) and Rongsayamanont et al. (2017). The properties of the foamate and cell-free broth are summarized in Table 1.

**TABLE 1:** Properties of surfactants.

**Parameter**	**Biosurfactant**			**Dehydol LS7TH**
	**Foamate (pH 7)**	**Cell-free broth (pH 7)**	**Cell-free broth (pH 10)**	
Head group	Anionic			Non-ionic
Classification	Lipopeptides			Fatty alcohol C_12__–__14_ with 7 moles ethoxylate
Concentration	10.9 g/L	6.4 g/L	6.4 g/L	99.7^%a^
CMC at 25°C	0.3 g/L^b^	1.4 g/L^b^	1.2 g/L	0.02 g/L
Surface tension at CMC (mN/m)	28.4^b^	28.9^b^	28.5	29.8
CMD at 25^o^C (dilution times)	27.5^c^	22.7	21.8	ND
Surface tension at CMD (mN/m)	26.1^c^	28.6	27.6	ND

*^*a*^Data was provided from manufacturer. ^*b*^Data was obtained from Rongsayamanont et al. (2017). ^*c*^Data was obtained from Khondee et al. (2015). ND = no data, CMC = Critical micelle concentration; CMD = Critical micelle dilution.*

Dehydol LS7TH, a fatty alcohol C_12__–__14_ with 7 ethoxylate groups (99.7% purity), was purchased from the Thai Ethoxylate Co., Ltd., (Thailand). It is a non-ionic synthetic surfactant derived from palm oil. 1-butanol (99% purity, Fisher Scientific, United Kingdom Limited) was used as a hydrophilic linker. Chloroform (99%, +), 1-hexane (99%, +), methanol (99%, +), and dichloromethane (99%, +) were purchased from Sigma–Aldrich (Saint Louis, MO) and used as solvents. A saline solution [4% (w/v)] was prepared by dissolving 4 g sodium chloride (NaCl, 99%, +) in 100 mL deionized water. All other chemicals were of analytical grade.

Synthetic-based mud (SBM), drill cutting, and linear C_9_ to C_21_ α-olefins (LAOs, polyolefin) were obtained from an onshore oilfield in Thailand. Polyolefin is the main component in SBM. It was used as a surrogate for residual SBM present in the drill cutting. The drill cuttings were excavated from the onshore drilling operation with a well depth of 3,000–3,500 m from the surface. Particle diameter of the drill cuttings as determined by sieve analysis (ASTM D422) was smaller than 75 μm. The initial TPH concentration in the drill cuttings was 151,572 mg/kg cuttings (>15% w/w) (Table 4). Total hydrocarbons present in the drill cuttings were mainly asphaltenes (50.5% w/w) and saturated hydrocarbons (49.5% w/w).

*Marinobacter salsuginis* RK5 and *Microbacterium saccharophilum* RK15 were isolated from seawater as crude oil-degrading bacteria. They were maintained in Zobell Marine Broth 2216 (HiMedia Laboratories) and deposited at the MSCU culture collection, Thailand Bioresource Research Center (TBRC), as MSCU 1054 and 1055, respectively. *Marinobacter salsuginis* RK5 had a low cell hydrophobicity (8%) but a high EPS producing activity (95%), while both cell hydrophobicity and EPS producing activity of *Microbacterium saccharophilum* RK15 were high at 78% and 97%, respectively. To increase their polyolefin-degrading activity, they were mixed with a fuel oil-degrading bacterium, *Gordonia amicalis* JC11 (previously *Gordonia* sp. JC11), which had been studied for oil spill remediation in the presence of biosurfactants (Laorrattanasak et al., 2016). Marine bacteria were initially selected to avoid the salt stress due to the high conductivity of drill cuttings (Table 4). Biochar derived from wood and NPK fertilizer (30:5:5) were purchased from local agriculture companies.

### Development of the Bio-Based Washing Agent Using Microemulsion Phase Behavior Experiments

**FIGURE 1:**
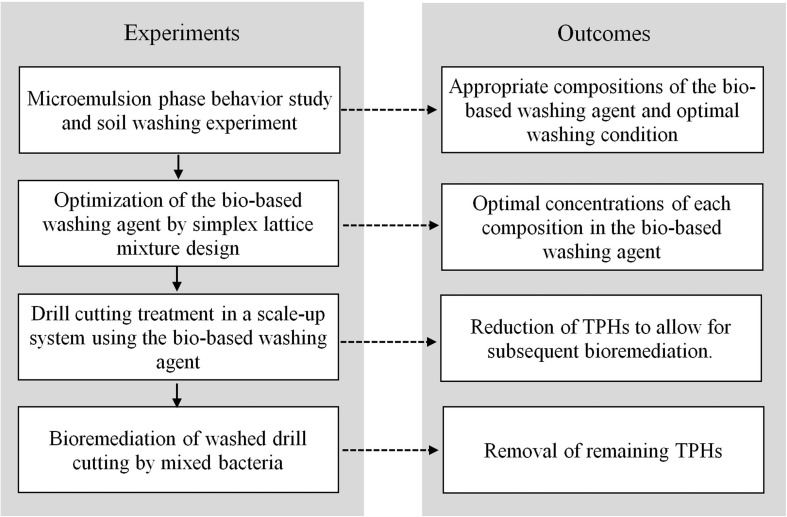
Flowchart of experimental procedure and outcomes.

The flowchart of experimental procedure and outcomes is presented in Figure 1. The development of bio-based washing agent was carried out using microemulsion phase behavior experiments to identify the appropriate compositions for an efficient formulation. Previously, researchers in our laboratory have applied the hydrophilic-lipophilic deviation (HLD) concept of binary anionic surfactant mixtures to formulate crude oil spill dispersants by mixing lipopeptide biosurfactants with sodium dihexyl sulfosuccinate (Rongsayamanont et al., 2017). However, no HLD concept exists for a mixture of anionic and non-ionic surfactants. Therefore, in this study a bio-based washing agent based on microemulsion phase behavior experiments was formulated. The microemulsion phase experiments were performed in 1 mL flat-bottom glass vials with PTFE caps. This method was adapted from Tongcumpou et al. (2003). In this study, 0.2 mL of polyolefin and 0.8 mL of the surfactant solution with different saline concentrations were added to the vials. The surfactant solution contained varying concentrations of foamate, Dehydol LS7TH and butanol. All samples were mixed with a vortex mixer for 1 min and then left to reach equilibrium at 25°C for 24 h. After the systems reached equilibrium, the relative phase volumes were quantified for each sample to determine the microemulsion type. The phase structure and characteristics of each microemulsion have been reported in literature (Salager et al., 2013; Arpornpong et al., 2018). The dynamic interfacial tension (IFT) of the SBM and bio-based washing formulations were measured by a spinning drop tensiometer (SVT 20, Dataphysic Instruments).

### Soil Washing Experiment in a Small System and Jar Tests

In a small system test, ten grams of dried drill cutting was suspended in a vial containing 20 mL of the bio-based washing agent. The suspension was then mixed for 30 min by placing on an orbital shaker at 200 rpm. The sample was centrifuged for 30 min at 3,000 rpm to separate the TPHs from the drill cutting and the washing agent. The drill cutting was rinsed twice with distilled water to remove the remaining TPHs and washing solution. The TPHs remaining in the washed cutting were determined to calculate the TPH removal efficiency over the initial TPHs in drill cuttings.

A jar test experiment was conducted to investigate the impacting factors on TPH removal of the selected formulations under varying washing conditions. The washing conditions were varied by altering the washing time and the cuttings-to-washing agent ratio. Initially, 100 grams of dried SBM drill cutting was loaded into a 1 L glass beaker containing different volumes of washing agent (ranging from 200–400 mL) to achieve cuttings (g) to washing agent (mL) ratios of 1:2, 1:3, and 1:4. In the jar test, the suspension was then mixed at a speed of 150 rpm for 5–30 min. Further washing steps were performed using the same washing procedure as in the small system test. All washing experiments were performed in triplicates.

### Optimization of the Bio-Based Washing Agent for the Scale-up System

Statistica 8.0 software was used to obtain the optimum formulation of simplex lattice mixture comprising lipopeptides, Dehydol LS7TH, and water, for the semi-pilot scale experiment. The concentration ranges for lipopeptide and Dehydol LS7TH solutions were selected based on the microemulsion phase behavior experiments. A 14-run design matrix was generated (Table 3) and a triangular surface with three factors, three polynomial degrees and augmented with interior points was constructed. The TPH removal efficiency from batch washing experiments was the response variable in the experimental design. Each experiment was performed in duplicates. The most suitable mathematical fitting model was selected by comparing various statistical parameters provided by the one-way analysis of variance (ANOVA), such as *p*-value, lack of fit and *R*-squared value (Visetvichaporn et al., 2020). The selected model was then applied to predict the suitable formulation of the bio-based washing agent.

Since the production of foamate by a large-scale foam fractional process is difficult and rather costly, cell-free broth obtained from the production process was applied instead of foamate. The pH of the cell-free broth was adjusted to 10 with NaOH. Batch washing experiments were conducted in vials. The cuttings were vigorously mixed with the washing solution in a vortex mixer for 10 min. The cuttings-to-liquid ratio was fixed to 1:4 based on the results of the jar test experiment. The washing solution was separated from the cuttings via centrifugation at 5,000 rpm for 10 min. The washed cuttings were rinsed twice with deionized water. The TPHs remaining in the washed cutting was determined to calculate the TPH removal efficiency over the initial TPHs in the cuttings.

### Drill Cutting Treatment Process in the Scale-up System

The scale-up washing process for the removal of TPHs from cuttings consisted of a rotary screener, hydrocyclone, agitation mixing tank and rotary vacuum-drum filter. The formulation of the bio-based washing agent and the cuttings-to-liquid ratio were obtained from the mixture design and jar test experiments, respectively. The maximum capacity of this process was 40 kg/day. The drill cuttings were transferred into the rotary screener to remove any coarse bits of gravel (>10 mm) at a flow rate of 40 L/min. The fined cuttings were mixed with the washing solution in the hydrocyclone and then transported to the agitation mixing tank, which operated at a contact time of 30 min and agitation rate of 250 rpm. The washing solution was separated from the cuttings using the rotary vacuum-drum filter at a rotation rate of 0.6 rpm with water spraying, which was repeated twice. The TPHs remaining in the washed cuttings were determined to calculate the TPH removal efficiency over the initial TPH in the cuttings.

### Polyolefin Biodegradation Experiment

After the washing process, residual TPHs and bio-based washing components might remain in the drill cuttings. This study therefore investigated the polyolefin degradation efficiency of mixed *Marinobacter salsuginis* RK5, *Microbacterium saccharophilum* RK15, and *Gordonia amicalis* JC11 in the presence of the bio-based washing solution. The polyolefin biodegradation test was conducted in a 125 mL flask containing 45 mL MSM medium, which consisted of 2.5 g/L NH_4_Cl, 5.46 g/L KH_2_PO_4_, 4.76 g/L Na_2_HPO_4_, 0.20 g/L MgSO_4_, and 5.0 g/L NaCl (Arulazhagan and Vasudevan, 2011). The concentration of NaCl was lower than that of seawater because the drill cuttings attained a decreased conductivity after washing (Table 4). The concentration of polyolefin in the medium was varied at 0.1, 0.25, and 0.5% (v/v), while the bio-based washing formulation was added based on a dispersant-to-oil ratio (DOR) of 1:10. The DOR was adopted to represent the optimal amount of washing formulation when applied to disperse the polyolefin on the water surface and promote its solubilization. To prepare the bacterial inoculum, each strain was cultivated in Zobell Marine Broth and incubated at room temperature with shaking at 200 rpm. The 24 h culture of each bacterial inoculum was harvested, adjusted to a concentration of 10^9^ CFU/mL and mixed at an equal volume. The mixed inoculum was added to the medium at 5 mL/flask, while the control experiment consisted of an uninoculated sample for determining the polyolefin loss by abiotic processes. All experiments were conducted in triplicates and incubated with shaking at 200 rpm. The concentration of residual TPHs and the bacterial number were analyzed from the flasks sacrificed on days 0, 7, and 14.

### Bioremediation of Washed Drill Cutting Experiment

Bioremediation of the washed drill cutting was performed in a soil microcosm under aerobic condition. Prior to bioremediation, the washed drill cutting samples were dried and ground to 2 mm particles. Their soil texture, conductivity, organic matter content, bacterial number and available nutrients were characterized using standard methods. The washed drill cuttings had a silty clay texture with a low nutrient level and small bacterial number (Table 4). Consequently, mixed bacterial inoculum, fertilizer and biochar were applied for bioremediation to promote TPH biodegradation and improve soil properties. There were five treatments: (1) unamended, (2) biochar, (3) biochar and mixed bacteria, (4) biochar and fertilizer, and (5) biochar, fertilizer and mixed bacteria. In each treatment, the washed drill cuttings (soil) were mixed with the amendments to obtain a total weight of 200 g, and then placed in a 9 × 14 × 5 cm^3^ plastic box with a solid lid. The proportion of soil and biochar was 20% (w/w) or 50% (v/v), while the fertilizer was applied to achieve a C:N:P ratio of 100:10:1. The moisture content of the inoculated and uninoculated microcosms was adjusted to 25% WHC by adding bacterial inoculum and distilled water, respectively. The mixed bacterial inoculum was prepared as in the polyolefin biodegradation experiment. The soil microcosm experiments were conducted in triplicates and incubated at room temperature. The boxes were opened every week, and the soil was manually mixed to provide aeration, while the moisture content was adjusted by adding distilled water. On days 0, 7, 21, 35, and 49, soil samples (6 g each) were collected from two locations in the box and mixed well before determining the TPH concentration and bacterial number.

### Analytical Methods

The amount of TPHs in the drill cuttings was analyzed by thin layer chromatography-flame ionization detector (TLC-FLD, with an Iatroscan^TM^ MK-6/6S, Mitsubishi Kagaku Iatron, Inc., Japan) and gas chromatography-flame ionization detector (GC-FID, with a Hewlett-Packard 6890, Agilent Technologies, United States) in the washing and bioremediation experiments, respectively. The TPHs were extracted from the cuttings by mixing twice with chloroform at a ratio of 2:1 and the chloroform was separated from the cutting via centrifugation at 5,000 rpm for 20 min. The concentration of TPHs in the chloroform was analyzed by TLC-FID and GC-FID according to Khondee et al. (2012). The concentrations of TPHs were calculated based on a standard polyolefin curve.

The surface tension, critical micelle concentration (CMC) and critical micelle dilution (CMD) were determined with a digital tensiometer (Kruss, K10ST, Germany) at 25°C via the plate method. The CMC was obtained from the cross-section of the plot between the surface tension and concentration of crude lipopeptides, while the CMD was obtained from the cross-section of the plot between the surface tension and serial dilution of the cell-free supernatant.

The bacterial number in the polyolefin biodegradation experiments was determined on Zobell Marine agar because the bacteria were previously cultivated in MSM medium containing NaCl. During bioremediation of the washed drilled cuttings, the total bacteria were counted on tryptone soya agar (TSA) to include all the soil heterotrophic bacteria, while the oil-degrading bacteria were counted on NSW agar overlaid with 50 μL polyolefin. The bacteria were dislodged from the soil by sonicating the soil suspension for 2 min followed by 1 min of vortex mixing. The process was repeated twice, and serial dilutions were then prepared before plate counting.

## Results

### Development of the Bio-Based Washing Formulations

Microemulsion phase behavior experiments can reveal different microemulsion types depending on the composition of the aqueous and oil phases. From literature, surfactant solutions can form different types of microemulsions (Type I, III, and II) by varying the concentrations of cosurfactant (Hayes et al., 2013), lipophilic linker (Bourrel et al., 1980; Salager et al., 2013; Phaodee et al., 2018), salinity (Acosta and Bhakta, 2009; Arpornpong et al., 2018; Phaodee et al., 2018) or temperature (Salager et al., 2013; Arpornpong et al., 2018) in the system. The phase behavior of polyolefin with either lipopeptide biosurfactant (as foamate) or Dehydol LS7TH indicated the occurrence of microemulsion Type I (Supplementary Table 1). However, the mixtures of foamate and Dehydol LS7TH generated Type I to III microemulsions with polyolefin when the concentrations of NaCl or butanol increased (Supplementary Figures 1, 2). The phase transition was due to the increase in hydrophobicity of the system. The minimum amount of Dehydol LS7TH required in the surfactant mixture to form Type III microemulsion was 2% (v/v) (Supplementary Figure 3).

**FIGURE 2:**
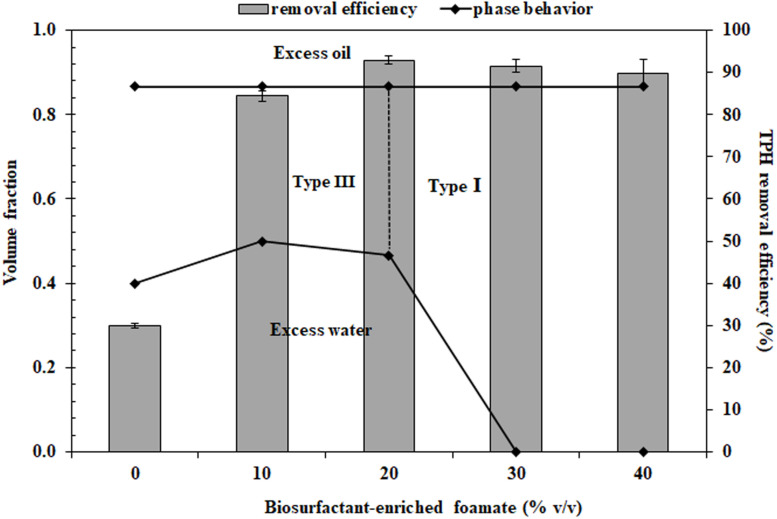
Microemulsion phase behavior of polyolefin and surfactant solution containing 2% Dehydol LS7TH, 4% butanol and varying volumes of foamate in saline water. The petroleum hydrocarbon removal efficiency of each formulation was conducted with drill cutting in a batch experiment. The ratio of cutting (g) to washing agent (mL) was 1:2 and the washing time was 30 min.

To formulate the bio-based washing agents, the volume of foamate was varied to achieve lipopeptide concentrations below and above the CMC. Figure 2 shows the phase behavior and TPH removal efficiency of the bio-based washing agents at various foamate concentrations containing 2% Dehydol LS7TH and 4% butanol in saline water. Phase transition was observed from Type III to I with increasing foamate concentration. This occurs because the increased foamate concentration leads to a decrease in hydrophobicity of the system. A similar trend has been observed for anionic surfactants with a strong hydrophilicity (Wade et al., 1978). According to Figure 2, the TPH removal efficiency sharply increased as the foamate concentration increased in the system containing 2% Dehydol LS7TH and 4% butanol in saline water. The greater TPH removal was observed as the foamate concentration increased beyond its CMD value (Table 1). The efficiency reached its highest value of 92.9 ± 1.0% at a foamate concentration of 20% or approximately 5x CMD. The concentration of foamate at 20% was therefore selected to formulate bio-based washing agents (F1 to F5 formulations).

Due to complex mixtures of drill cuttings (e.g., cutting, drilling mud, inorganic and organic matters), they originally contained some amount of dissolved salts (e.g., Ca^2+^, Mg^2+^, and Na^+^). NaCl or CaCl_2_ was added to the drilling mud as an alkalinity controller and brine enhancer during the drilling process. Calcite (CaCO_3_), hydrophilite (CaCl_2_) and halite (NaCl) are the main components found in the drill cuttings as reported in literature (Filippov et al., 2009; Mansour et al., 2016; Poyai et al., 2020). The initial salinity of drill cutting was indirectly measured using an electrical conductivity meter. The electrical conductivity (EC) of the drill cuttings was 4,290 μs/cm (∼0.5% (w/v) salinity). It was thus assumed that the salinity of the drill cutting would not significantly affect the total concentration of NaCl in the bio-based washing agents. The role of DI and saline water (4% w/v NaCl) in bio-based washing agents were compared in the washing experiment.

The TPH removal efficiency increased from 50.3% (only foamate) and 61.9% (only Dehydol LS7TH) to 99.2% (20% foamate and 2% Dehydol LS7TH in formulation F1) (Table 2). The efficiency of F1 to F5 formulations containing 20% foamate and 2% Dehydol LS7TH as the major components were compared and evaluated based on the ability to form microemulsion and decrease the residual TPHs in the drill cuttings. Many investigations reported that the formations of microemulsion Type I (near the Type I to III transition) and Type III can be applied for enhanced petroleum recovery (Childs et al., 2005), site remediation (Wu et al., 2000; Quraishi et al., 2015), and detergency (Phaodee et al., 2018). The most efficient formulation, F1, produced a Type I microemulsion with polyolefin and contained only 20% (v/v) foamate and 2% (v/v) Dehydol LS7TH in water. The F3 formulation contained similar surfactant mixtures and produced a Type I microemulsion as with the F1 formulation. However, it had a significantly lower TPH removal efficiency than that of the F1 formulation, which was probably due to the use of saline water. On the other hand, the effect of saline water was different in the formulations containing butanol. The F2 formulation (forming Type III microemulsion in saline water) had higher TPH removal efficiency than the F4 formulation (forming Type I microemulsion in water). When the amount of butanol in the formulations increased, i.e., from 4% butanol in formulation F2 to 8% butanol in formulation F5, the TPH removal efficiency decreased. However, these formulations produced a microemulsion Type III with polyolefin and attained a low dynamic interfacial tension (Supplementary Figure 4).Thus, microemulsion formation is not the only parameter governing oil removal efficiency, but other parameters also play a role in oil washing.

**TABLE 2:** Effectiveness of bio-based washing agents on removal of petroleum hydrocarbons from drill cuttings in a batch experiment.

**Formulation^a^**	**Composition**	**Microemulsion type^b^**	**TPH Removal efficiency^c^ (%)**
–	Deionized water (DI)	Not occur	29.9 ± 0.6
–	Foamate (20% v/v)	Type I	50.3 ± 1.0
–	Dehydol LS7TH (2% v/v)	Type I	61.9 ± 1.3
F1	20% Foamate + 2% Dehydol LS7TH + 78% DI	Type I	99.2 ± 0.2
F2	20% Foamate + 2% Dehydol LS7TH + 4% Butanol + 74% saline water	Type III	92.9 ± 1.0
F3	20% Foamate + 2% Dehydol LS7TH + 78% saline water	Type I	74.8 ± 2.2
F4	20% Foamate + 2% Dehydol LS7TH + 4% Butanol + 74% DI	Type I	81.2 ± 6.0
F5	20% Foamate + 2% Dehydol LS7TH + 8% Butanol + 70% saline water	Type III	78.6 ± 0.7

*^*a*^Compositions of each formulation were reported as volume of each component. The saline water contains 4% (w/v) NaCl. ^*b*^Microemulsion type was tested with polyolefin. ^*c*^Ratio of cutting (g) to washing agent (mL) was 1:2 and washing time was 30 min.*

### Application of the Bio-Based Washing Formulations in Jar Tests

**FIGURE 3:**
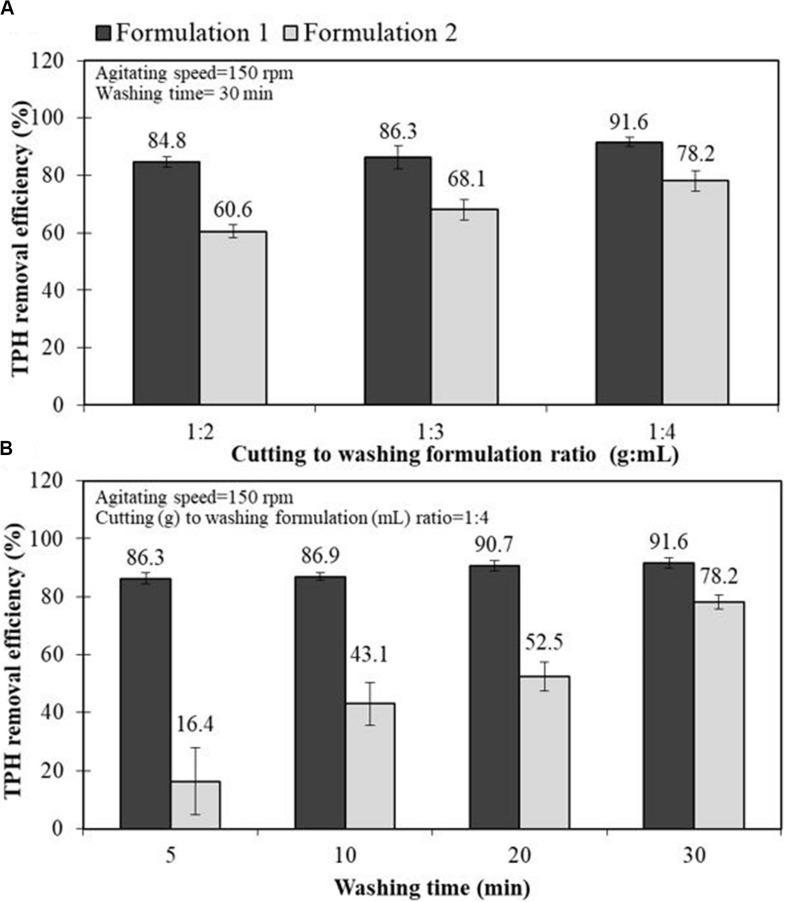
Effect of drill cutting-to-washing agent ratio **(A)** and washing time **(B)** on the petroleum hydrocarbon removal efficiency of the bio-based washing formulations, F1 (20% Foamate + 2% Dehydol LS7TH + 78% DI) and F2 (20% Foamate + 2% Dehydol LS7TH + 4% Butanol + 74% saline water) in a jar test experiments.

For a scale-up experiment, the effects of the drill cuttings-to-washing agent ratio and washing time on the performance of the two selected bio-based washing agents in removing TPHs from the drill cuttings were investigated via jar tests. The F1 and F2 formulations were chosen based on their high oil removal efficiency and they were representatives of the formulations that generated microemulsion Type I and III, respectively. Figure 3A shows the TPH removal efficiency as a function of the cuttings-to-washing agent ratio. The results show that when the washing agent loading of the system was increased, the TPH removal efficiency tended to increase. This occurs because the system contains sufficient surfactant to penetrate the cuttings with less coalescence between the surfactant and oil (Naksuk et al., 2009). The cuttings-to-washing agent ratio of 1:4 had the maximum TPH removal efficiency (up to 91.6%). Figure 3B reveals that a washing time of 30 min for the cuttings was considered the optimum time for the TPHs to detach from the cuttings and solubilize into the micelles. The results exhibit a similar trend for both formulations. As expected, the F1 formulation provided a higher TPH removal efficiency than the F2 formulation. These results were consistent with those of the previous experiments in this study.

### Optimization of the Bio-Based Washing Formulation for the Scale-up System

According to the small-scale study, the F1 formulation containing only lipopeptide and Dehydol LS7TH was appropriate for bio-based washing agent. The formulation is simple but the cost might be high due to the high concentration of each composition. Consequently, the F1 formulation was further optimized via the simplex lattice mixture design to obtain an efficient formulation with lower amounts of surfactants. In addition, cell-free broth containing lipopeptides was used instead of foamate to avoid difficulties associated with biosurfactant recovery via foam fractionation process. The initial concentration of lipopeptides in the cell-free broth was 3.0 g/L, and its surface activity was enhanced by adjusting the pH to 10 (Table 1). The Dehydol LS7TH solution was prepared at 5% (v/v).

A range of cell-free broth and Dehydol LS7TH concentrations were prepared based on the results from the microemulsion phase behavior experiments; therefore, 16.70–100.00% cell-free broth (pH = 10) and 0.84–5.00% Dehydol LS7TH were chosen. Table 3 lists the experimental plan from the simplex lattice mixture design and the obtained responses for each run. The empirical coefficient values and ANOVA outcomes, as well as the statistical criteria for the responses, are presented in the Supplementary Data. The maximum *R*-squared value of the full cubic regression model demonstrated the best fit between the biobased washing agent compositions (independent variables) and TPH removal (dependent variable) compared to other models (Supplementary Table 2). The lack of fit *p*-value of higher than 0.05 confirms the applicability of this model. The fitted full cubic equation is as follows:

**TABLE 3:** Simplex lattice mixture design of three components and results of response values for optimizing of the F1 formulation.

**Mixtures**	**Independent variables (%)**			**Dependent variables (TPH removal efficiency, %)**
	**Cell-free-broth, pH 10 (100%)**	**Dehydol LS7TH (5%)**	**Water (100%)**	
1	100.0	0.0	0.0	44.2
2	0.0	100.0	0.0	21.0
3	0.0	0.0	100.0	30.2
4	33.3	66.7	0.0	78.4
5	33.3	0.0	66.7	44.1
6	0.0	33.3	66.7	46.8
7	66.7	33.3	0.0	53.8
8	66.7	0.0	33.3	79.9
9	0.0	66.7	33.3	54.1
10	33.3	33.3	33.3	81.0
11	66.7	16.7	16.7	74.2
12	16.7	66.7	16.7	55.6
13	16.7	16.7	66.7	53.1
14	33.3	33.3	33.3	75.8

TPHremoval(%)=43.619X+ 21.185Y

+ 29.441⁢Z+ 145.122⁢X⁢Y+ 120.076⁢X⁢Z

+ 108.118⁢Y⁢Z+ 84.404⁢X⁢Y⁢Z- 153.632⁢X⁢Y⁢(X-Y)

(1) + 195.865⁢X⁢Z⁢(X-Z)-3.866⁢Y⁢Z⁢(Y-Z)

where X = cell-free broth (lipopeptide 0.3%, pH 10), Y = water and Z = Dehydol LS7TH (5%).

**FIGURE 4:**
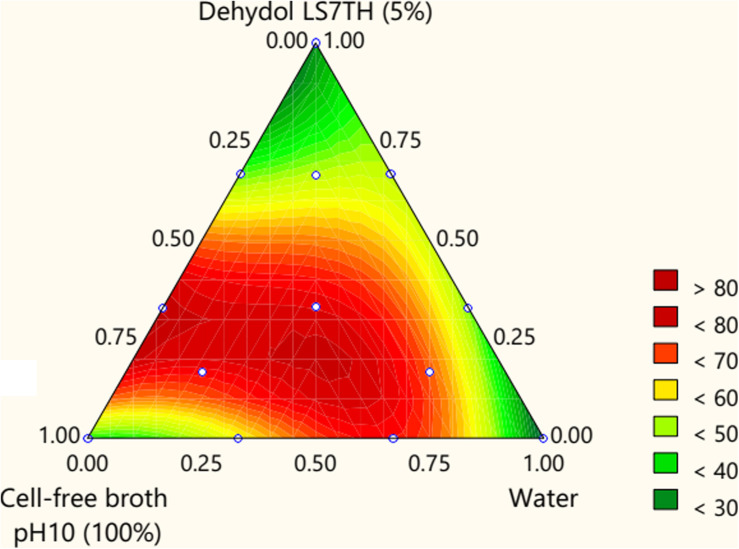
Contour response surface plot for optimizing the components of F1 formulation. Lipopeptide solution was used as cell-free-broth (pH = 10). The initial concentration of Dehydol LS7TH was 5% (v/v). The ratio of cutting (g) to washing agent (mL) was 1:4.

A sequential *p*-value below 0.05 indicates that the model terms are statistically significant. Both the cell-free broth and Dehydol LS7TH have a significant effect on the TPH removal efficiency (Supplementary Table 3). The coefficients X to Z are positive, indicating the synergistic effect of the lipopeptide and Dehydol LS7TH mixture. The optimal formulation of the bio-based washing agent consisted of 72.0% cell-free broth and 1.4% Dehydol LS7TH in water (Figure 4). The predicted response from this optimal formulation was a TPH removal efficiency of 81.2%. The developed bio-based washing agent was applied to a scale-up washing experiment for TPH removal from cuttings. The obtained TPH removal efficiency was 70.7% at an initial TPH concentration of 151,572 mg/kg (Table 4). The loss of washing capability likely occurred during the scale-up process. For example, polyolefins were redeposited with the cutting particles during the separation of washing solution from the washed cuttings using the rotary vacuum-drum filter.

**TABLE 4:** Characteristics of drill cuttings after washing in a scale-up system and after mixing with biochar.

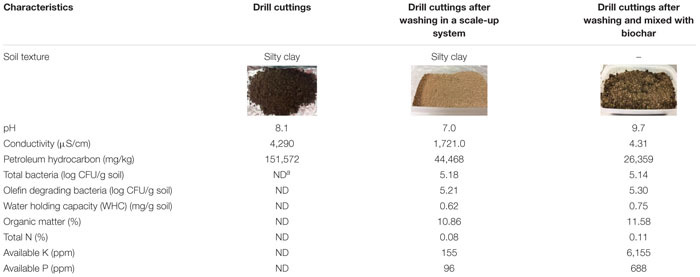

*^*a*^ND = not determined.*

### Biodegradation of Polyolefin by the Mixed Bacteria

**FIGURE 5:**
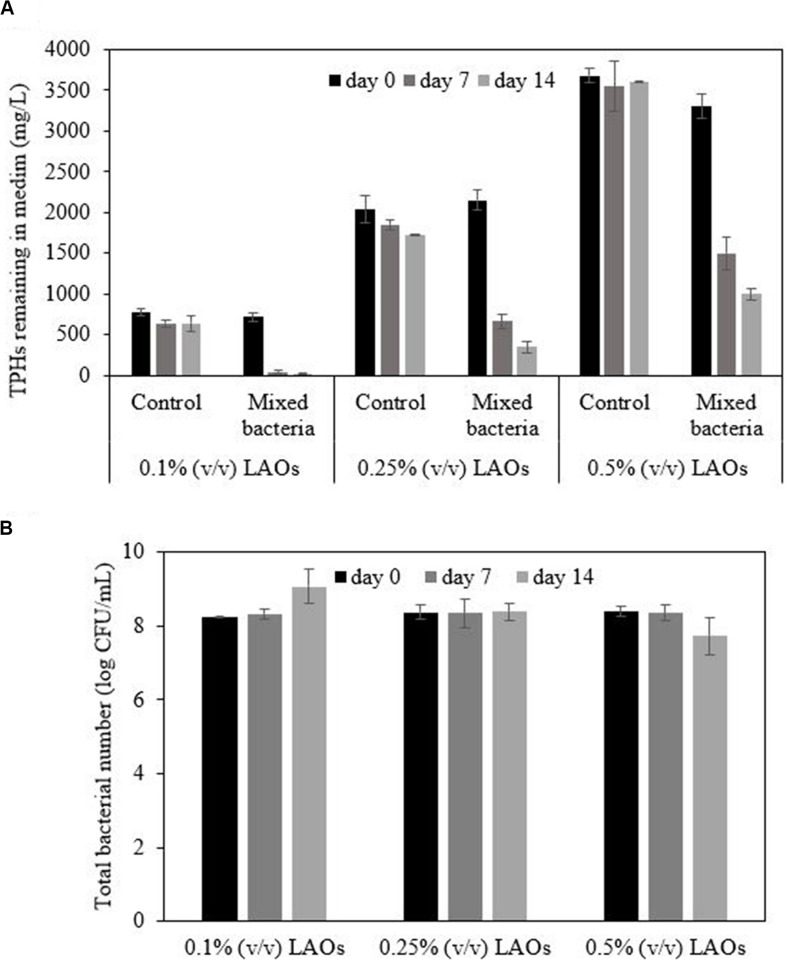
Degradation of polyolefin (LAOs) by mixed bacterial inoculum in MSM medium containing 0.5% NaCl **(A)** and the changes in bacterial number during the experiment **(B)**.

*Marinobacter salsuginis* RK5, *Microbacterium saccharophilum* RK15, and *Gordonia amicalis* JC11 slightly degraded polyolefin when applied as a single strain (data not shown). The mixture of these bacteria was expected to impose a synergistic effect on the polyolefin biodegradation efficiency because each strain should have different characteristics and catabolic pathways. The LAOs in all inoculated samples decreased over time. After 14 days, the mixed bacterial inoculum showed the highest removal efficiency with 0.1% LAOs (97%), followed by 0.25% LAOs (84%) and 0.5% LAOs (70%), while the control treatment only attained a removal efficiency of 9–18% (Figure 5A). The bacterial number in the 0.1% LAO system increased by approximately 1 magnitude to 9.07 log CFU/mL at the end of the study, corresponding to a decrease in the LAOs (Figure 5B). On the other hand, the bacterial number in the 0.25% LAO system remained constant at 8.38 log CFU/mL, while it was slightly reduced to 7.73 log CFU/mL in the 0.5% LAO system. The low cell numbers might be due to the formation of aggregated cells, as indicated by the orange cell clumps in the 0.25% LAO system and the attached film in the 0.5% LAO system (Supplementary Figure 5), which interfered with the dilution of bacterial cells during the plate count technique. The difference in cell aggregation could be due to the presence of the bio-based washing formulation, which was applied according to the amount of oil in each flask. The different concentrations of biosurfactant molecules might promote different forms of aggregation of polyolefin, bacterial cells and bacterial metabolites. The presence of viable cells suggested that longer incubation time could increase the biodegradation efficiency of the mixed bacterial inoculum. Since the mixed bacterial inoculum was able to efficiently degrade low concentrations of polyolefin, they were applied for bioremediation of the washed drilled cuttings.

### Bioremediation of the Washed Drill Cuttings

**FIGURE 6:**
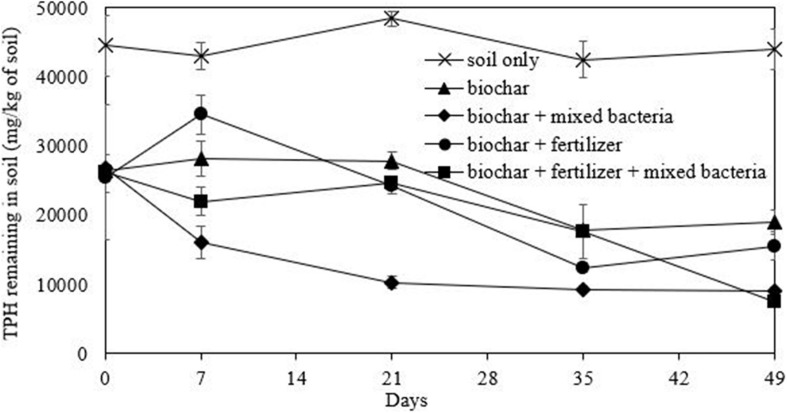
Efficiency of bioremediation approaches comprising of mixed bacterial inoculum, biochar and fertilizer on the removal of petroleum hydrocarbons (TPHs) from drill cuttings after washing in a scale-up system.

In this study, biochar was initially mixed with the washed drilled cuttings to improve soil characteristics such as water holding capacity and available nutrients (Table 4). The biochar*-*amended washed drill cuttings had a notable decrease in conductivity from 1,721 to 4.31 μS/cm, while the available potassium and phosphorus increased from 155 and 96 ppm to 6,155 and 688 ppm, respectively. The concentration of the TPHs in biochar*-*amended washed drill cuttings decreased by approximately half (from 44,468 mg/kg to 26,359 mg/kg) due to the dilution effect (Table 4). Other characteristics, such as bacterial number and organic matter and total nitrogen contents, in the biochar*-*amended washed drill cuttings were similar to those of the initial sample. The unamended sample or the natural attenuation treatment attained only 1% TPH removal efficiency, and TPHs remained at a concentration of 43,989 mg/kg after 49 days of incubation (Figure 6). On the other hand, treatment combining biochar, fertilizer and mixed bacteria achieved the highest TPH removal efficiency of 71%. This TPH removal efficiency was calculated from the TPH concentrations on day 0 and day 49, which were 26,359 and 7,515 mg/kg, respectively (Figure 6). The second most efficient treatment was the biochar- and mixed bacteria-amended microcosms, which removed 66% of the TPHs with 8,968 mg/kg of TPHs remaining at the end of experiment. Without bacterial addition, the removal of TPHs was inefficient, of which the biochar-fertilizer treatment and biochar treatment removed 39 and 28% of TPHs, respectively. The results indicated that the addition of mixed bacteria played an important role in TPH removal from the biochar-amended washed drill cutting. The changes in alkane composition as revealed by the GC-FID chromatograms on day 49 also confirmed that the TPHs were significantly reduced by both physical and biological mechanisms in the treatments containing mixed bacteria (Supplementary Figure 6).

**FIGURE 7:**
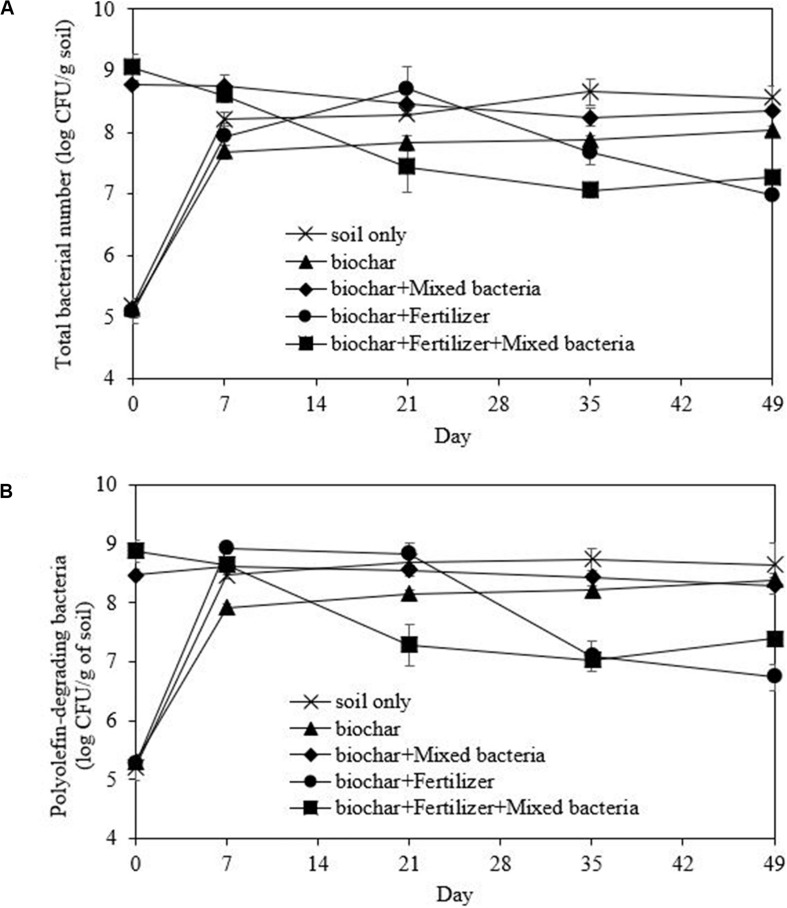
Changes in number of total bacteria **(A)** and polyolefin-degrading bacteria **(B)** during the bioremediation of drill cuttings after washing in a scale-up system.

In all microcosms, the growth curves of both total and olefin-degrading bacteria showed a similar trend, but the numbers of olefin-degrading bacteria were slightly lower at each time point (Figures 7A,B). The initial washed drilled cuttings had a low bacterial number; thus, the addition of mixed bacteria was necessary. The rapid decrease in TPHs in the biochar and mixed bacteria treatment (Figure 6) corresponded with the numbers of total and olefin-degrading bacteria after incubation, which were maintained throughout the incubation period at 8.51 and 8.47 log CFU/g soil, respectively (Figures 7A,B). The addition of fertilizer also promoted the growth of indigenous bacteria in the washed drill cuttings from day 0 to 21; however, they later competed with the added bacterial inoculum (Figure 7) and led to delayed TPH degradation (Figure 6). The average bacterial number in the treatment with biochar, fertilizer and mixed bacteria decreased to 7.33 log CFU/g soil on day 49, which was lower than that in the treatment with only biochar and mixed bacteria. Competition between different indigenous populations might also occur since the bacterial numbers in biochar and fertilizer treatment were lower than that in the treatment with biochar only. However, the number of indigenous olefin-degrading bacteria in the treatments with no bacterial addition increased from an average of 5.25 log CFU/g soil to 7.92 log CFU/g soil at the end of the study (Figure 7B). The TPH removal efficiencies in the uninoculated microcosms, i.e., soil only, biochar and biochar-fertilizer treatments were low to moderate (Figure 6). The results indicated that the TPH-degrading activity of the indigenous olefin-degrading bacteria was lower than that of added mixed bacterial inoculum. Consequently, bioaugmentation with mixed bacterial inoculum was appropriate for the treatment of washed drill cuttings.

## Discussion

Microemulsion phase behavior studies were conducted to develop suitable bio-based washing formulations. The formation of microemulsions between foamate (highly hydrophilic character) and polyolefin (highly hydrophobic character) implies the attainment of a low interfacial tension and the possibility of substantial oil solubilization. This occurs because of the balance between the hydrophilic water and hydrophobic oil phases. The best washing formulation F1, comprised of 20% foamate and 2% Dehydol LS7TH in water, could form Type I microemulsion with polyolefin (near the transition region between Type I and Type III). The high TPH removal efficiency by Type I microemulsion might be due to the super-solubilization effect, where the swollen micelles incorporate a large amount of oil into their hydrophobic core (Tongcumpou et al., 2003; Javanbakht and Goual, 2016). The same trend was found in the study by Wu et al. (2000), who reported that the microemulsion Type I (near the Type I to III boundary) and Type III had similar oil solubilization capacities. Since there was no butanol or salt in formulation F1, it was considered a bio-based and environment-friendly washing agent.

The present study found that butanol reduced the TPH removal efficiency of formulations (Formulation F2, F4, and F5). One possibility is that butanol reduces the micellar cavity where the SBM and polyolefin prefer to solubilize in micelles. The results obtained in this study correspond to those reported by Bourrel et al. (1980) and Tongcumpou et al. (2003), whereby an increased lipophilic linker of the system can decrease oil solubilization. Moreover, saline water was not required in the bio-based washing formulation. The presence of salt and cation (Na^+^) probably influenced anionic surfactant adsorption on drill cuttings and precipitation between Na^+^ and foamate anionic surfactant. Increase in surfactant adsorbed onto cutting, lead to an increased surfactant loss during the washing process. The effect of salinity on anionic surfactant adsorption at the solid–liquid interface has been discussed by many researchers (Childs et al., 2005; Arpornpong et al., 2010; Belhaj et al., 2020). In addition, Arpornpong et al. (2018) showed scanning electron microscopy images of spent bleaching earth (SBE) surface before and after surfactant-based washing procedure and found that a high salinity washing solution (15% w/v NaCl) increased the adsorption of ethoxylate non-ionic surfactant on SBE surface.

The TPH removal efficiency of surfactant mixtures was higher than either lipopeptides or Dehydol LS7TH alone. These results confirmed that the higher efficiency was due to the synergistic effect. Shi et al. (2015) also reported that the mixed anionic–non-ionic surfactants considerably enhanced the oil solubilization capacity and interfacial tension reduction, which resulted in a decrease in the total amount of surfactant used in a particular application, which in turn reduced both the cost and the environmental impact. A single-biosurfactant-based washing agent usually attains less than 90% TPH removal from drill cutting. For example, Yan et al. (2011) reported that the maximum TPH removal efficiency of oil-based mud from drill cuttings was 83% at a rhamnolipid concentration up to 360 mg/L, a solid:liquid ratio of 1:3 and a 20 min washing time, while Olasanmi and Thring (2020) attained an 85.4% TPH reduction from drill cuttings with a rhamnolipid solution (500 mg/L) at an optimal agitation rate of 100 rpm for 30 min and a ratio of cutting-to-washing agent of 1:1.

When formulation F1 was applied in the jar tests, the results indicated an optimal washing condition consisting of a 30 min washing time at a drill cutting to bio-based washing agent ratio of 1:4. However, the efficiency of formulation F1 was reduced to 91.6% due to the large amount of drill cutting in a jar test, which caused a reduction in the mixing speed and redeposition of TPHs during solid-liquid phase separation. The same trend was found in other studies (Kantar and Honeyman, 2006; Elgh-Dalgren et al., 2009). The jar test suggested that a large volume of formulation F1 was required for washing drill cuttings on site. To reduce the cost of formulation F1, the concentration of each component was optimized via the simplex lattice mixture design, and cell-free broth was used instead of foamate. The drawback of a lower lipopeptide concentration in cell-free broth than that in foamate was resolved by increasing the pH of cell-free broth solution from 7 to 10. Alkaline condition can reduce the adsorption of anionic surfactants on drill cuttings by increasing electrical negative charge on the surface of rock solids (Kurnia et al., 2020). Thus, the surfactant activity increased, resulting in a decrease in the amount of surfactant required in the bio-based washing agents. Moreover, the alkali might react with the acid components in SBM present in the cuttings to produce soap (Sheng, 2014). The presence of soap helps in the reduction of IFT, providing a greater oil mobilization from the cuttings during the washing process. Although the washing efficiency obtained with cell-free broth (81.2%) was lower than that obtained with foamate (91.6%), the TPH removal efficiency was comparable to that of other studies (Yan et al., 2011; Olasanmi and Thring, 2020). The application of non-purified lipopeptides in foamate and cell-free broth indicates an economic advantage of the developed bio-based washing agents for drill cutting treatment.

The bio-based washing agents can be applied to other cleaning applications, for example, washing of petroleum-contaminated soil. The formulation can be further optimized by increasing the salt or hydrophobic surfactant concentration for heavier crude oil-contaminated soil. For saline soil with different salinity and hardness levels, the washing agent can be formulated by adjusting the salt concentration or adding appropriate amounts of builder or lime-soap dispersing agent. Childs et al. (2005) evaluated the performance of surfactant-based washing agents for the treatment of oil-based drill cuttings. They found that the addition of C_8_-sulfobetaine, a lime soap dispersing agent (LSDA), and sodium metasilicate (Na_2_SiO_3_), a builder, with a small amount of added salt in the surfactant-based washing agent, could enhance the oil removal efficiency from drill cuttings. For a long storage period, potassium sorbate preservative maybe added to the bio-based washing agents, as reported by Freitas et al. (2016), to maintain their activities.

To date, there have been few studies on the scale-up of drill cutting treatment by the surfactant washing technique. Elgh-Dalgren et al. (2009) attained a lower PAH removal by soil washing at the pilot scale than that attained at the laboratory scale due to the variation in environmental conditions such as the ambient temperature. This research also found a decrease in TPH removal from 81.2% at the laboratory scale to 70.7% at the semi-pilot scale. Therefore, the combination of biosurfactant washing and subsequent treatment, such as bioremediation was required to enhance the TPH removal from the drill cuttings obtained from the oilfield. Considering the cost of wastewater management due to the surfactant washing approach, the recovery and reusability of bio-based washing solutions should be studied further. Nevertheless, the biological treatment of wastewater containing bio-based washing solutions should be easier than that of wastewater containing chemical-based washing solutions due to its low toxicity, high biodegradability, and high biocompatibility of lipopeptide biosurfactants.

The residual TPHs in the bio-based washing solutions as well as in the washed drilled cuttings should be biodegraded before disposal. Polyolefin, the main component in SBM, is a mixture of unsaturated hydrocarbons with medium chain lengths. It is relatively persistent under natural conditions. The degradation of olefins in a marine anaerobic biodegradation test lasted longer than 9 months of incubation and required sediment containing a large number of sulfate reducing bacteria, general anaerobes and methanogens as a source of inoculum (Herman and Roberts, 2005). Solid-phase tests revealed that synthetic drilling fluids containing olefins are slowly degraded in mud and coarse sand by indigenous microorganisms, where their half-life increases as the nominal concentration increases (Munro et al., 1998). Non-etheless, bioremediation can increase the olefin biodegradation rate. Approximately 96% of the 14,720 mg/kg linear alpha-olefin in a subsoil sample was reduced by providing a nitrogen source under aerobic condition, and the treated soil after 93 days of incubation showed low toxicity to plants, earthworms and microorganisms (Visser et al., 2002). It is thus possible to further enhance polyolefin biodegradation by adding efficient bacterial inoculum under aerobic condition. This study found that the mixture of *Marinobacter salsuginis* RK5, *Microbacterium saccharophilum* RK15, and *Gordonia amicalis* JC11 was able to degrade the polyolefin in the liquid medium containing a biobased washing agent. The uses of mixed bacteria probably promote the success of bioaugmentation because they have complementary catabolic pathways as well as the ability to enhance the oil dispersion and hydrocarbon bioavailability (McGenity et al., 2012). The extent of polyolefin removal in the bioaugmented sample was much higher than that of the abiotic process. However, the degrading activity of mixed bacteria decreased at high polyolefin concentrations (>2.5%); thus, the bacterial inoculum was applied for bioremediation of drill cuttings only after the washing process.

Bioremediation of washed drill cuttings was examined in a microcosm study with varying amendments for 49 days. The results indicated that only mixed polyolefin-degrading bacteria and biochar were required for an enhanced TPH removal from the washed drill cuttings. Although the above treatment resulted in more remaining TPHs (0.89%) than that with additional fertilizer (0.75%), it attained a higher biodegradation rate, and its cost was relatively lower. Biochar enhanced the activity of the mixed bacterial inoculum probably by providing a habitat for microorganisms and improving the bulk density, pH and movement of air, water and nutrients within the soil matrix (Gul et al., 2015). In addition, biochar can sorb petroleum hydrocarbons and their degrading intermediates, which leads to a considerable reduction in soil toxicity and allows continuous petroleum biodegradation (Qin et al., 2013). The presence of biosurfactant molecules in the washed drill cuttings might also increase the bioavailability of TPHs. Brown et al. (2017) reported that biochar and rhamnolipid biosurfactant imposed a synergistic action on enhancing oil biodegradation in soil during landfarming. During bioremediation, the number of added bacteria decreased over time, probably due to their inability to compete with indigenous bacteria. Zhang et al. (2016) reported that biochar-immobilized *Corynebacterium variabile* HRJ4 achieved high TPH biodegradation, which made the bioremediation process more robust against environmental factors and other competitors. Thus, the bacterial inoculum should be immobilized on biochar before application to washed drill cuttings.

The developed sequential bio-based washing and bioremediation system removed TPHs in the drill cuttings. The TPH concentration in the drill cuttings was reduced from 151,572 mg/kg to 8,968 mg/kg, i.e., TPH removal efficiency of 94.1%. The efficiency of this system was comparable to that of Yan et al. (2011), where oil-based drill cuttings with an initial TPH concentration of 85,000 mg/kg were washed with a rhamnolipid solution and then subjected to bioremediation by adding sawdust, as a bulking agent, and mixed bacterial culture. They found that the concentration of TPHs decreased to 5,470 mg/kg, i.e., 93.5% TPH removal efficiency (Yan et al., 2011). Consequently, the sequential remedial system could be applied in onshore drilling operations. It is expected that the sequential bio-based washing and bioremediation system will have lower cost and environmental impacts than the current incineration technique. The treated drill cuttings contained less than 1% TPHs and could be further treated on site by rhizoremediation. The technology uses synergistic interactions of plant roots and rhizospheric microorganisms to degrade hydrocarbons and is considered one of the most suitable treatment methods for petroleum-polluted soils over a large area (Hussain et al., 2018).

## Conclusion

Drill cuttings with high concentrations of TPHs (>15%) were sequentially treated with a bio-based washing agent and bioremediation. The formulation of the bio-based washing agents was guided by the experimental phase behavior of the mixed bio-based surfactants and polyolefin and further optimized via experimental design. The efficient formulation contained only lipopeptides and Dehydol LS7TH in water, which is considered environmentally friendly and cost effective. When the bio-based washing agents were applied in a large-scale system, the TPH washing efficiency decreased. It is thus necessary to treat the remaining TPHs by bioremediation. From a microcosm study, only mixed polyolefin-degrading bacteria and biochar were required for an enhanced TPHs removal from washed drill cuttings. The sequential remedial system can be applied on site without high energy use in contrast to incineration. It is also possible to use the bio-based washing agents for cleaning petroleum-contaminated soil and other polluted environments.

## Data Availability Statement

All datasets presented in this study are included in the article/Supplementary Material.

## Author Contributions

NA designed and formulated the bio-based washing agent. RP investigated biodegradation and bioremediation experiments. NK produced biosurfactant and optimized the bio-based washing agent. CT, SS, and KS supervised and conceptualized the bio-based washing formulations and washing systems. NA, NK, and EL analyzed the data and wrote the manuscript. EL designed the overall project, acquired funding and performed manuscript editing. All authors contributed to the preparation of manuscript.

## Conflict of Interest

The authors declare that the research was conducted in the absence of any commercial or financial relationships that could be construed as a potential conflict of interest.
