# Supplementary material for: Formulation of Bio-Based Washing Agent and Its Application for Removal of Petroleum Hydrocarbons From Drill Cuttings Before Bioremediation
Source: Front Bioeng Biotechnol. 2020 Aug 11;8:961. doi: 10.3389/fbioe.2020.00961 (PMC7431657; doi:10.3389/fbioe.2020.00961)
Supplement: Supplementary file 1 [file Data_Sheet_1.docx]

Supplementary Material


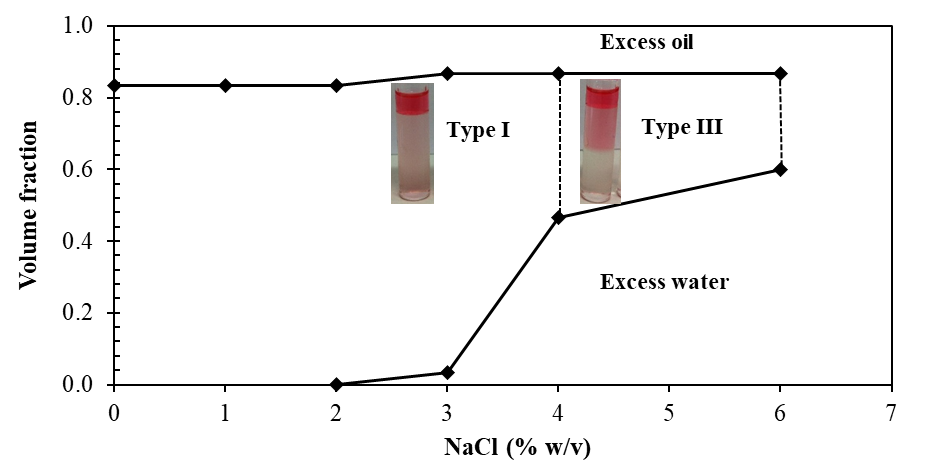


Supplementary Figure 1. Effect of NaCl concentrations in saline water on the microemulsion phase behavior between surfactant solution and polyolefin at 25°C. The surfactant solution contains 20% Foamate, 5% Dehydol LS7TH, 8% butanol and 67% saline water.

Supplementary Table 1. Types and physical characteristics of microemulsions.

| Aqueous phase | Oil phase | Microemulsion Type | Phase characteristic |
| --- | --- | --- | --- |
| Deionized water (DI) | Polyolefin | Not occur | **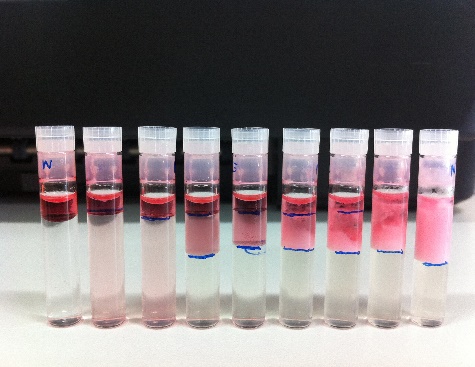** |
| Foamate (20% v/v) | Polyolefin | Type I | 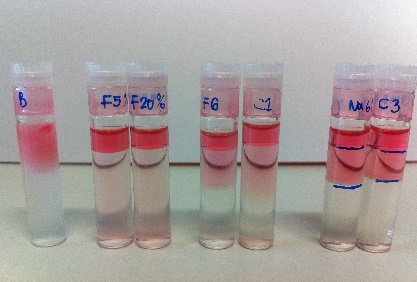 |
| Dehydol LS7TH (20% v/v) | Polyolefin | Type I | **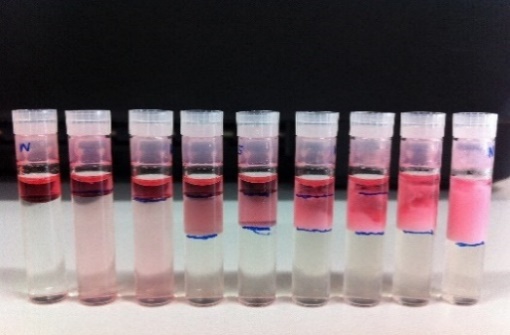** |
| Formulations* |  |  |  |
| F1, 20% Foamate + 2% Dehydol LS7TH + 78% DI | Polyolefin | Type I | 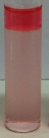 |
| F5, 20% Foamate + 2% Dehydol LS7TH + 8% Butanol + 70% saline water | Polyolefin | Type III | **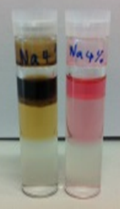** |
| F5, 20% Foamate + 2% Dehydol LS7TH + 8% Butanol + 70% saline water | Synthetic-based mud | Type III | **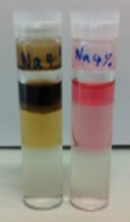** |

*The compositions of each formulation were reported as volume of each component. The saline water contains 4% (w/v) NaCl.


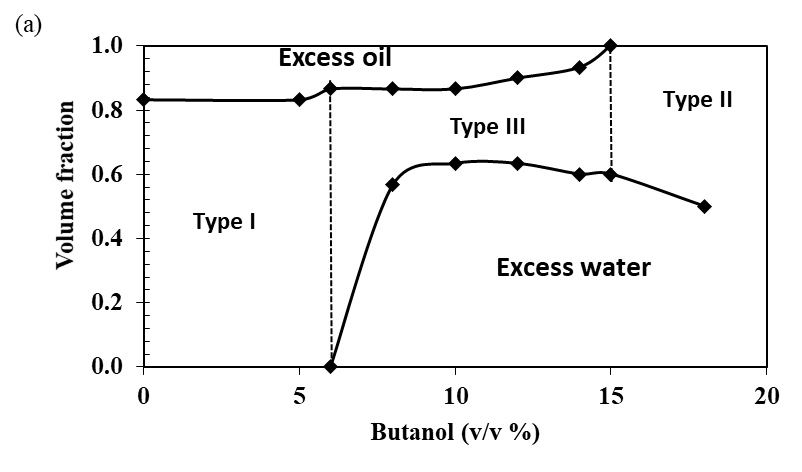


Supplementary Figure 2. The effect of butanol concentrations on the microemulsion phase behavior between surfactant solution and polyolefin at 25 °C. The surfactant solution contains 20% foamate and 5% Dehydol LS7TH with butanol scan.

**
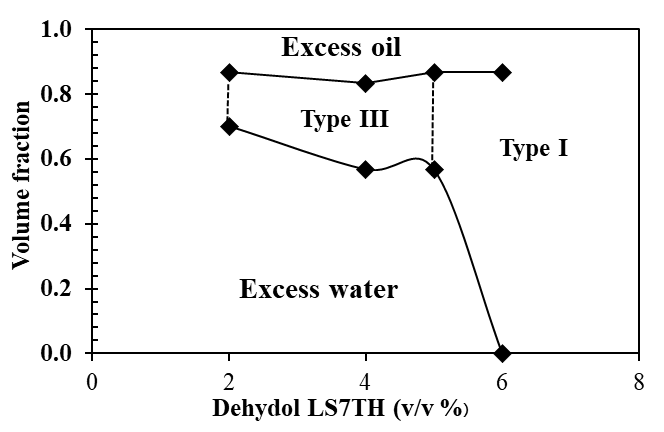
**

Supplementary Figure 3. The effect of Dehydol LS7TH concentrations on the microemulsion phase behavior between surfactant solution and polyolefin at 25 °C. The surfactant solution contains 20% foamate, 8% butanol and saline water with Dehydol LS7TH scan.


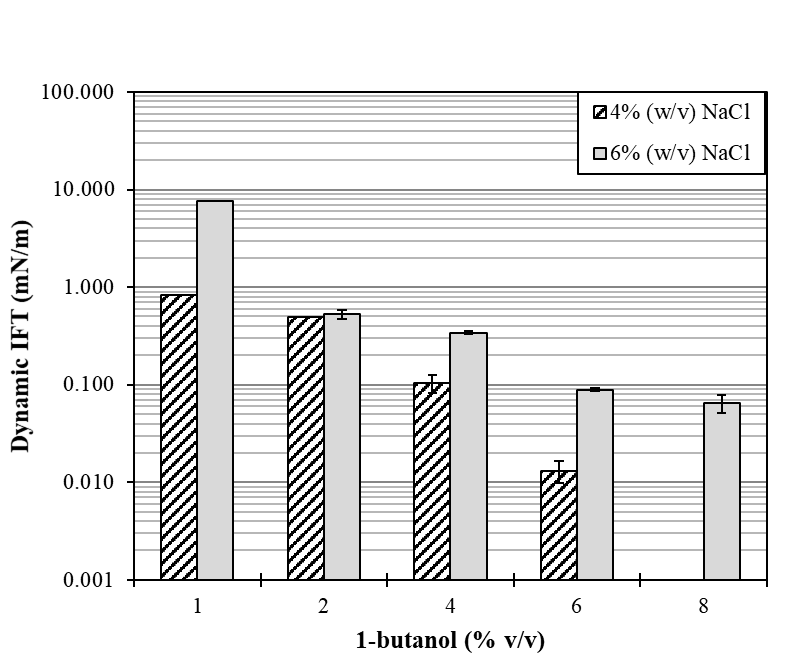


Supplementary Figure 4. Dynamic interfacial tension (IFT) of synthetic-based mud and surfactant soltutions containing 20% foamate and 5% Dehydol LS7TH with various concentrations of butanol and NaCl at 25 °C. Without surfactant solution, the dynamic IFT of synthetic-based mud and DI water was 18.28 mN/m.

Supplementary Table 2. Model summary statistics for bio-based washing agent. Lipopeptide solution was used as cell-free broth (pH=10). Initial concentration of Dehydol LS7TH was 5% (v/v). The ratio of cutting (g) to washing agent (mL) was 1:2.

| Model | Cell-free-broth and Dehydol LS7TH mixture | | |
| --- | --- | --- | --- |
|  | Regression, R-Sq (%) | Adjusted regression, R-Sq (adj) % | Lack of fit, P-value |
| Linear | 0.1541 | 0.0003 | 0.1449 |
| Quadratic | 0.7673 | 0.6218 | 0.2274 |
| Special cubic | 0.7696 | 0.5721 | 0.2108 |
| Full cubic | 0.9385 | 0.8001 | 0.2771 |

Note: Confident of interval = 95%

Alpha = 0.05

Supplementary Table 3. Regression coefficients for bio-based washing components to the full cubic model for cell-free-broth and Dehydol LS7TH mixture. Lipopeptide solution was used as cell-free broth (pH=10). Initial concentration of Dehydol LS7TH was 5% (v/v). The ratio of cutting (g) to washing agent (mL) was 1:2.

| Term | Coefficient | P-Value |
| --- | --- | --- |
| (X) Cell-free broth pH10 (100%) | 43.619 | 0.0067 |
| (Y) Water | 21.185 | 0.6671 |
| (Z) Dehydol LS7TH (5%) | 29.441 | 0.0255 |
| XY | 145.122 | 0.0189 |
| XZ | 120.076 | 0.0343 |
| YZ | 108.118 | 0.0468 |
| XYZ | 84.404 | 0.7178 |
| XY(X-Y) | -153.632 | 0.0994 |
| XZ (X-Z) | 195.865 | 0.0527 |
| YZ(Y-Z) | -3.866 | 0.9597 |

Note: Confident of interval = 95%

Alpha = 0.05

Day 0

Day 7

Day 14

0.1% (v/v)

LAOs

0.25% (v/v)

LAOs

0.5% (v/v)

LAOs


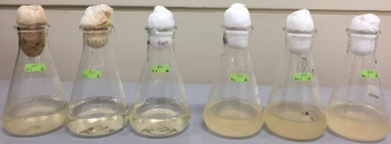

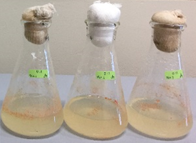

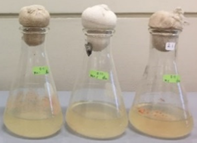


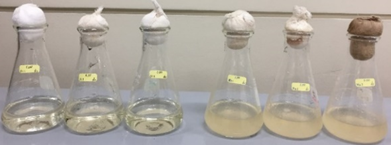

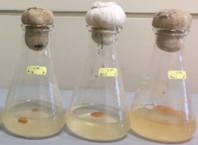

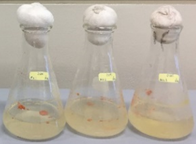


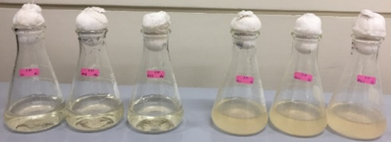

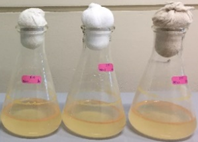

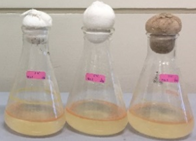


Control

Mixed bacteria

Supplementary Figure 5. Effect of LAOs concentrations on bacterial growth at day 7 and 14. Bacterial cells in orange color were clumped or attached to the surface of MSM medium, which might lead to the inaccuracy of plate count technique for bacterial number determination.


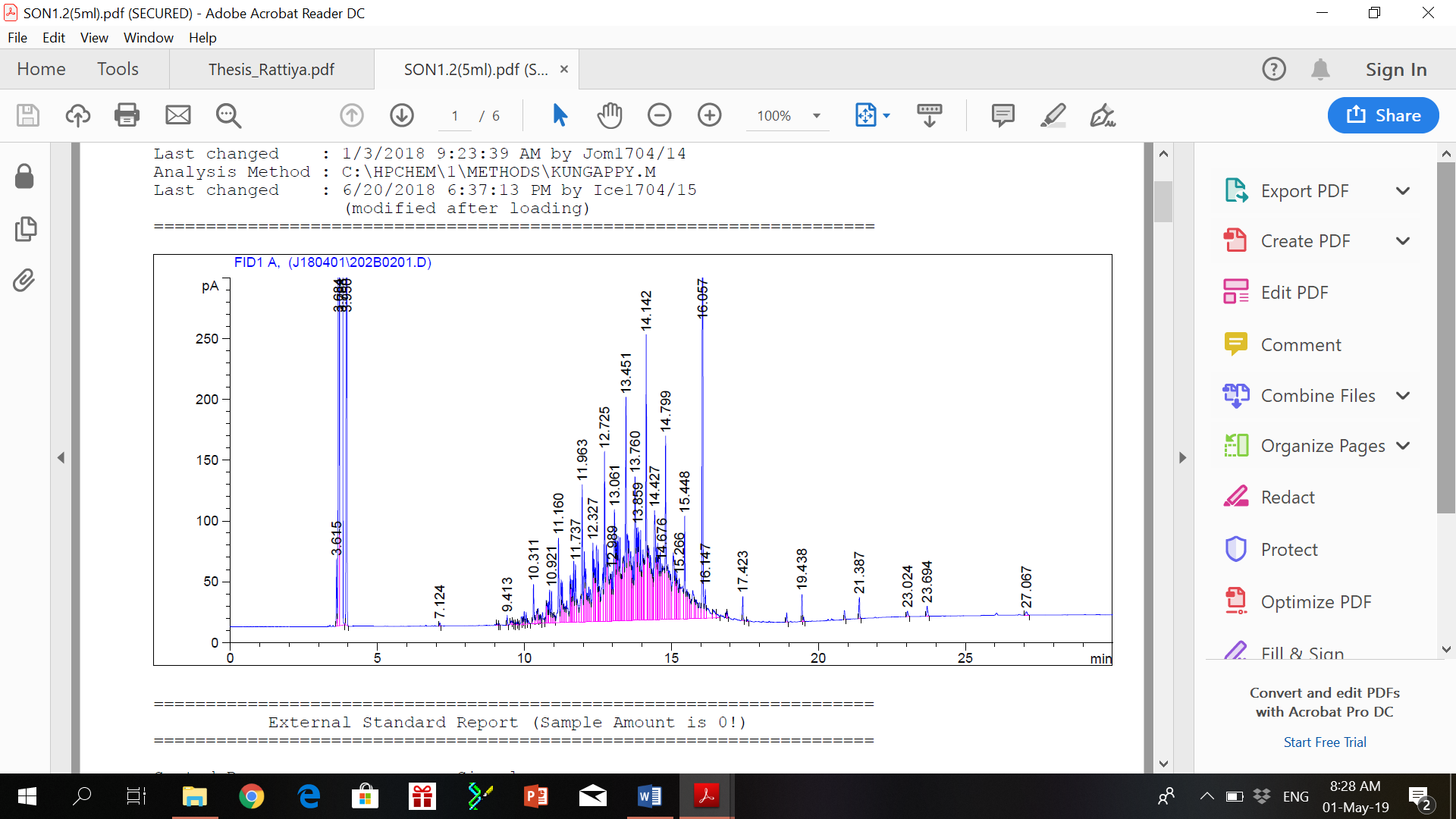


(A)

(B)


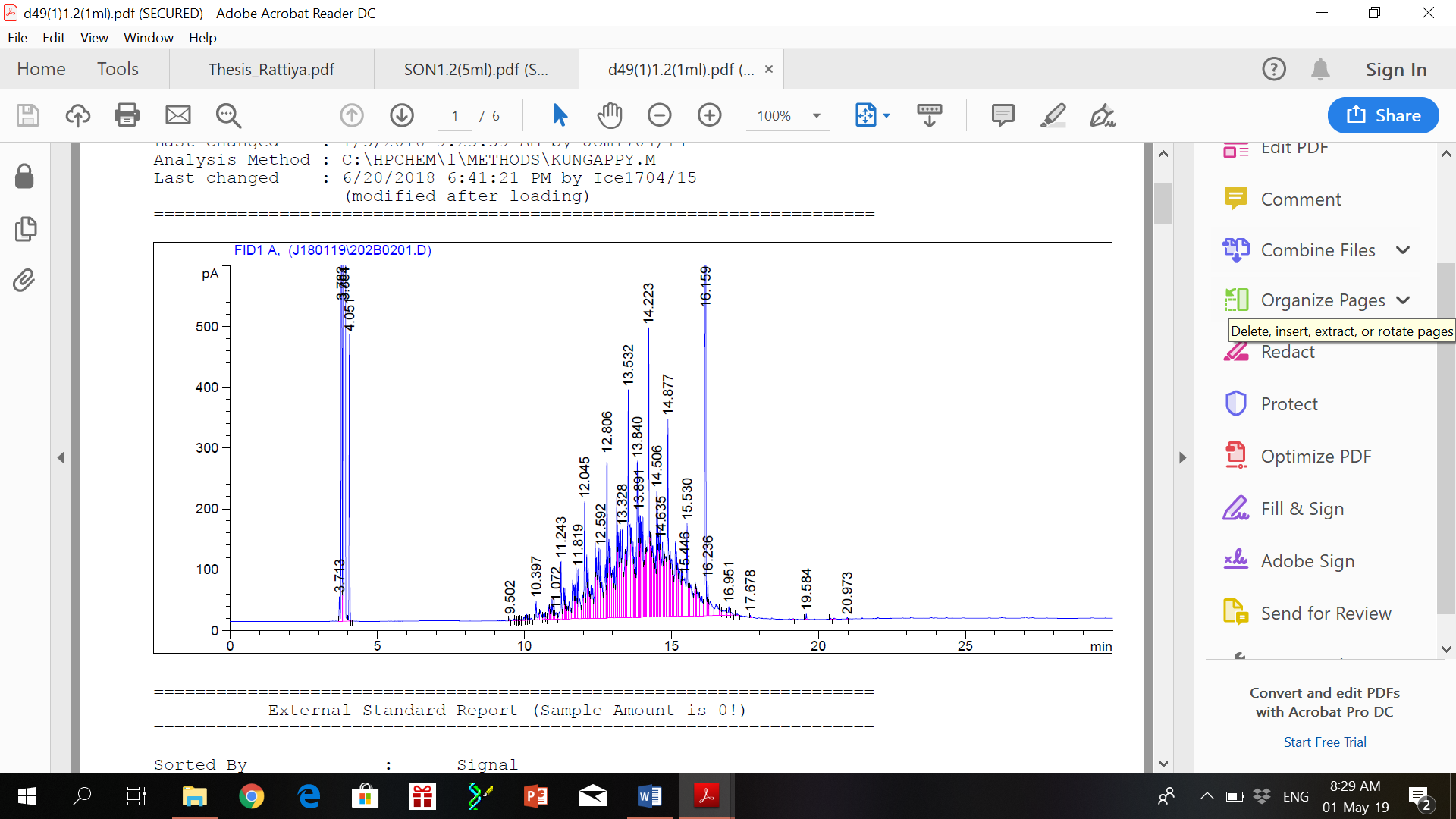


(C)


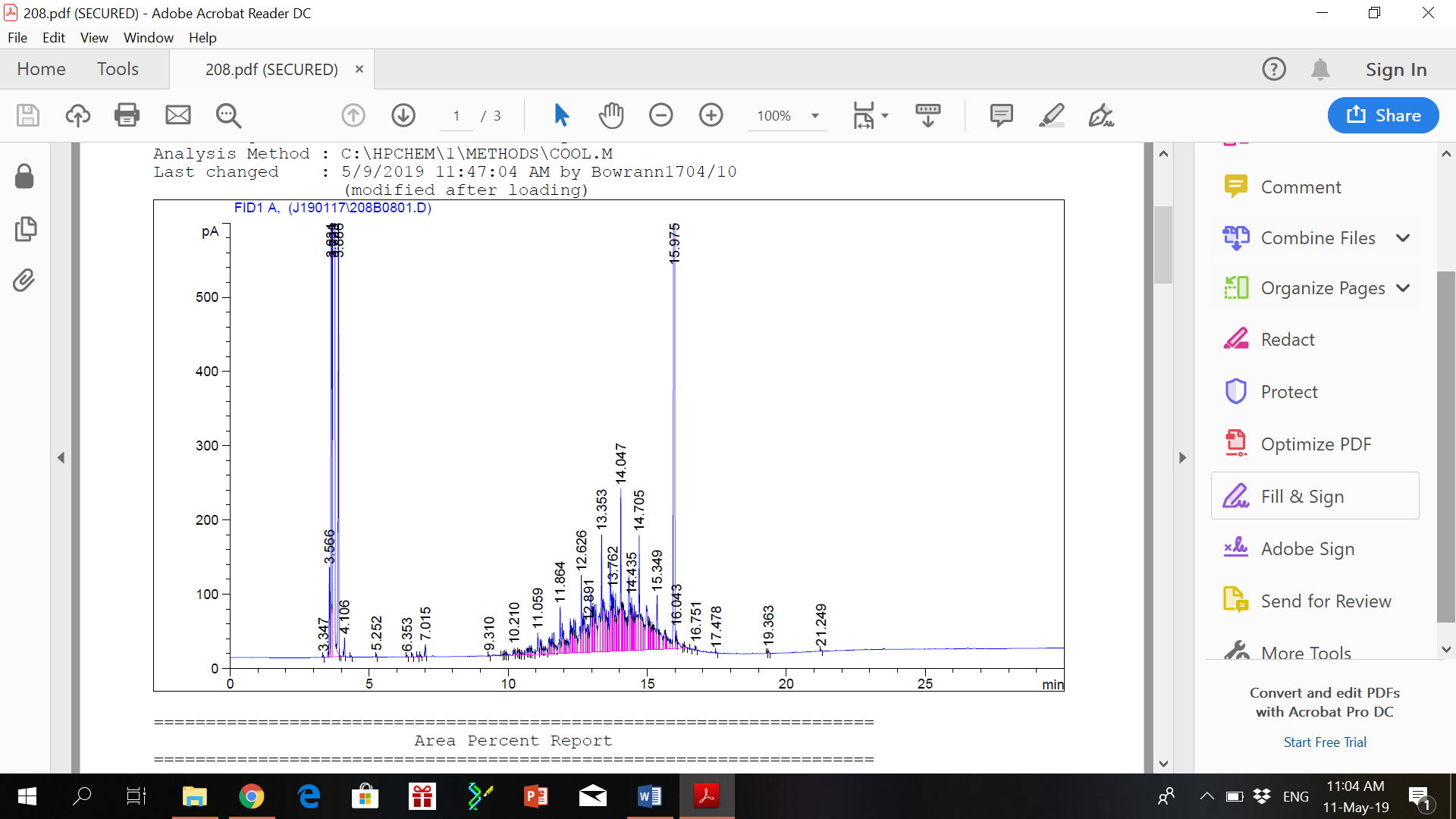


(D)


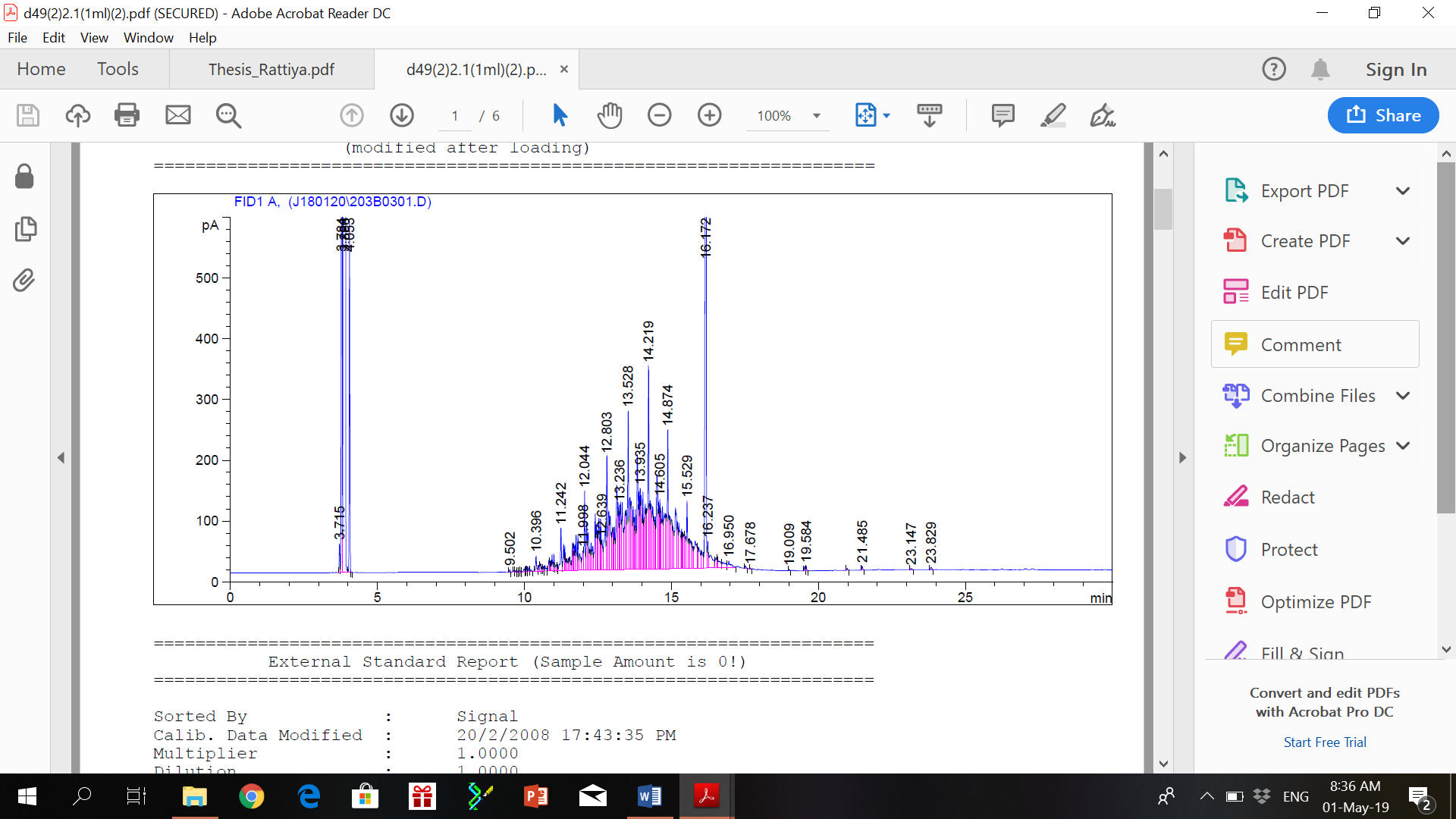


(E)


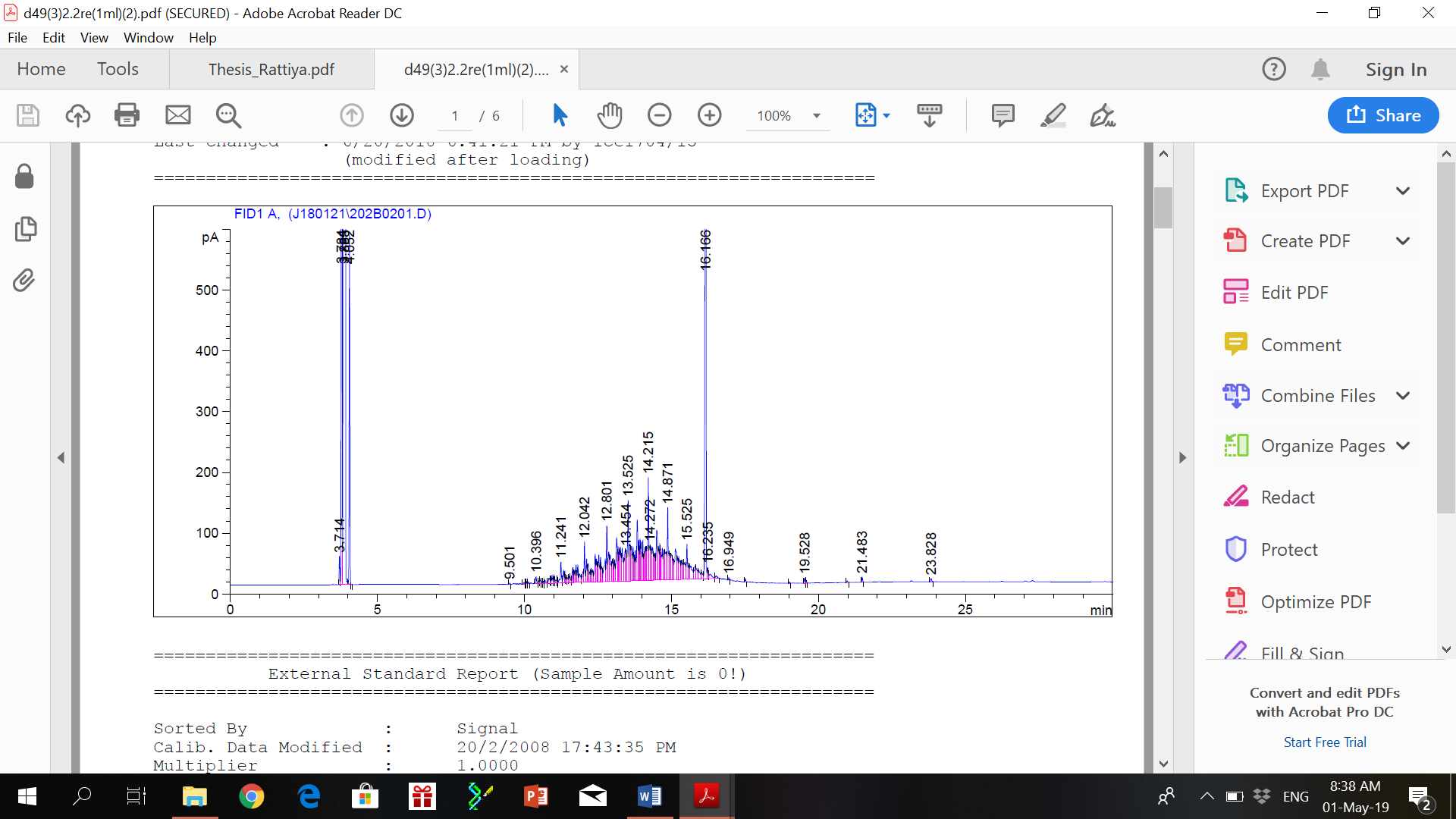


Supplementary Figure 6. GC chromatograms of TPH extracted from treated drill cuttings after day 49; A) soil only (the sample was diluted 5x before injecting to the GC-FID), B) biochar, C) biochar and mixed bacteria, D) biochar and fertilizer and E) biochar, fertilizer and mixed bacteria. The high peak at 15.9 - 16.2 min is internal standard.
